# Probiotic Consortia: Reshaping the Rhizospheric Microbiome and Its Role in Suppressing Root-Rot Disease of *Panax notoginseng*

**DOI:** 10.3389/fmicb.2020.00701

**Published:** 2020-04-30

**Authors:** Jinhao Zhang, Lanfang Wei, Jun Yang, Waqar Ahmed, Yating Wang, Lina Fu, Guanghai Ji

**Affiliations:** ^1^Key Laboratory of Agriculture Biodiversity for Plant Disease Management Under the Ministry of Education, Yunnan Agricultural University, Kunming, China; ^2^Agriculture and Rural Affairs Committee of Fengdu County, Chongqing, China

**Keywords:** *Panax notoginseng*, probiotic consortia, community composition, rhizosphere, microbiome structure and function

## Abstract

Root-rot disease caused by *Fusarium oxysporum* is a growing problem in agriculture for commercial cultivation of *Panax notoginseng.* Diverse microbes colonize plant roots, and numerous earlier studies have characterized the rhizospheric microbiome of *P. notoginseng*; nevertheless, the function of probiotic consortia on the rhizospheric microbiome against the root-rot disease remain elusive. We have compared and described the rhizospheric microbiome of lightly and severely diseased *P. notoginseng* as well as the interactions of the probiotic consortia and rhizospheric microbiome, and their function to alleviate the plant diseases were explored by inoculating probiotic consortia in bulk soil. From the perspective of microbial diversity, the rhizospheric dominant bacterial and fungal genera were utterly different between lightly and severely diseased plants. Through inoculating assembled probiotic consortia to diseased plant roots, we found that the application of probiotic consortia reshaped the rhizosphere microbiome, increasing the relative abundance of bacteria and fungi, while the relative abundance of potential pathogens was decreased significantly. We developed a microcosm system that provides a preliminary ecological framework for constructing an active probiotic community to reshape soil microbiota and restrain the disease. Microbial community structure differs between lightly and seriously diseased plants. The application of probiotic consortia changes the imbalance of micro-ecology to a state of relative health, reducing plant mortality. Plant disease suppression may be achieved by seeking and applying antagonistic microbes based on their direct inhibitory capability or by restructuring the soil microbiome structure and function.

## Introduction

Sanqi ginseng [*Panax notoginseng* (Burk.) F. H. Chen], a member of the Araliaceae family, holds a prominent position in traditional Chinese medicine in China and is high in demand (Lv et al., 2019). It grows at an altitude of 400–800 m above sea level under the shades of forests and at the slopes of mountains. It has several vital functions, including being hemostatic, blood stasis dissipating, and discutient, exhibiting acesodyne effects and antihypertensive, antithrombotic, anti-atherosclerotic, and neuroprotective actions (Yang et al., 2010, 2019; He et al., 2012; Guo et al., 2020). With the over-exploitation and predatory activities of wild ginseng, the available resources have become scarce. Therefore, the cultivation of *P. notoginseng* has gradually become the mainstream of the market. In China, the commercial cultivation of *P. notoginseng* began more than 200 years ago. However, the cultivation of *P. notoginseng* plants was hindered by replanting problems (Tan et al., 2017).

*Panax notoginseng* is a perennial plant, and if planted in a fixed plot for several years, it will reduce the quality and yield of tubers (Dong et al., 2013). Root-rot disease is the foremost hurdle of *P. notoginseng* due to its continuous cropping, the yield and quality of *P. notoginseng* are profoundly affected by this disease (Ma et al., 2013; Tan et al., 2017). Replanting of *P. notoginseng* has also failed due to low germination, poor seedling growth, and high seedling death rates (Yang et al., 2015). Soil-borne disease, nutrients deficiency, auto-toxicity, and the retrograde soil physicochemical properties are the factors that affect the replanting of *P. notoginseng* seedlings (Yang et al., 2019). Root-rot caused by the pathogen *Fusarium oxysporum* is a severe disease that inhibits the replantation of *P. notoginseng* plants (Dong et al., 2016). Many measures, such as chemical fungicides, soil modification practices, soil fumigation, and fertilizer application, are used to alleviate the replanting problem, but the results have not been satisfactory (Ou et al., 2012; Li et al., 2019).

Recently, using microbial antagonists as biological control agents has become an effective method to reduce the abundance of plant pathogens due to their non-toxic characteristics (Zheng et al., 2011). *Bacillus subtilis* XF-1 was inoculated into the clubroot affected Chinese cabbage rhizosphere, resulting in changing disease-suppressive microbial communities that can inhibit the abundance of *Plasmodiophora brassicae* Woron (Liu C.M. et al., 2018). The application of biocontrol bacteria could effectively alleviate the occurrence of root-rot. The *P. notoginseng* death rate and *Fusarium* abundance decreased by 63.3 and 46.1%, respectively, after inoculation with *B. subtilis* 50-1, which revealed that biocontrol uses microbial antagonists to alleviate replanting mortality (Dong et al., 2018). High bacterial diversity was associated with increased resistance to pathogen invasions and plant infestation, and the application of biofertilizers could also control the disease because biofertilizers contain beneficial microbes (Hu et al., 2016; Xiong et al., 2017). However, these studies have not reported the use of probiotic consortia containing several clear and simple strains to study the root microbiome and root-rot disease biocontrol on *P. notoginseng.* We selected the three most dominant genera (*Bacillus*, *Lysobacter*, and *Pseudomonas*) that have been reported as plants growth-promoting rhizobacteria (PGPR) or biocontrol bacteria and are frequently found in the rhizosphere of different crops (Postma et al., 2008; Postma and Schilder, 2015). For instance, *Bacillus velezensis* and *Lysobacter antibioticus* species are active antagonists of plant pathogens and are potential candidates as biocontrols for phytopathogens (Hayward et al., 2010; Xu et al., 2016; Martínez-Raudales et al., 2017). *L. antibioticus* produces lytic enzymes, a toxic compound that inhibits the growth of *Phytophthora capsici* and also restrains the DNA synthesis of the pathogen by producing a chemical named Myxin (Ko et al., 2009; Chowdhury et al., 2011). The growth of phytopathogenic Oomycetes (*Plasmopara viticola* and *Phytophthora infestans*) can be restricted by the secondary metabolites produced by *Lysobacter capsici* AZ78 (Puopolo et al., 2014, 2015). *Pseudomonas* spp. of bacteria is suitable for the colonization of plant roots, and many strains exhibit the activity of promoting plant growth or inhibiting the growth of various plant pathogenic bacteria (Liu K. et al., 2018).

Soil biodiversity is affected by the continuous cropping system, which implements a negative effect on soil health and productivity. Moreover, we can increase the impact of biodiversity by the introduction of rhizosphere microbial communities into the soil by adopting different methods (Berg and Smalla, 2009; Xiao et al., 2016). It is therefore important to understand the significance of soil microbial diversity for the continuous cropping of *P. notoginseng*. A study was carried out to investigate the response of rhizosphere and root endophytic bacteria under continuous cropping of *P. notoginseng*, and the highest bacterial diversity was found under healthy cropping system as compared to disease affected crops (Tan et al., 2017). A total of 279 bacteria were isolated through pure-culture methods from the rhizosphere soil of *P. notoginseng* plants, and it was revealed that 88 bacteria have an antagonistic activity to control root-rot disease (Fan et al., 2016). However, these studies only discussed the population and structure of the rhizosphere microbe of *P. notoginseng* without any microbiological treatment; this has thus sparked the hypothesis that changes in disease suppression are the joint effect of the application of microbes into the soil to inhibit the activity of pathogens or to balance the rhizosphere microbial diversity in the *P. notoginseng* cropping system (Vale et al., 2016). The bacterial community composition and diversity have been reported, and these have been found to be associated with disease suppression (Hamid et al., 2017; Mwaheb et al., 2017; Hussain et al., 2018).

To investigate this hypothesis, we used the root-rot of *P. notoginseng* caused by the soil-borne pathogens as a subject study. Therefore, we amended 3 years of continuous cropping field soil of *P. notoginseng* with probiotic consortia containing *Bacillus*, *Lysobacter*, and *Pseudomonas*, and we compared these treatments with no microbial inoculation (control) and biopesticide. We observed the impact of the four probiotic consortia on biocontrol efficacy, measured the growth characteristics and the quality (saponin contents and Sanqi root weight) of *P. notoginseng*, and also analyzed the bacterial and fungal community of *P. notoginseng* rhizosphere soil by Illumina MiSeq sequencing. These data provide an effective soil bioremediation method to alleviate the replanting failure of *P. notoginseng*. The results of this study also demonstrate how the probiotic consortia affect the rhizosphere soil microbiome for disease suppression. The aim of this study was to provide theoretical and experimental information to improve the structure and function of microbial communities to reduce the root-rot disease for continuous cropping of *P. notoginseng*.

## Materials and Methods

### Assembly and Study of Probiotic Consortia

*P. notoginseng* was used as a host plant to investigate the bacterial community associated with roots, using the biocontrol bacteria resources accumulated in the early stage of the laboratory. To evaluate the effectiveness of biocontrol bacteria, we selected previously verified pathogenic *F. oxysporum* as the target pathogen and tested their antagonistic activity against the mycelial growth of *F. oxysporum* on the potato dextrose agar (PDA) medium. We used 13 bacterial strains (Supplementary Table S1) to obtain four simplified probiotic consortia (Supplementary Table S2). Before the experiments, a single colony of each strain was selected and grown overnight in nutrient agar (NA: glucose or sucrose 10 g; peptone 5 g; beef extract 3 g; yeast extract 1g; agar 18 g; distilled water 1000 ml; pH 7.0). Each bacteria strain culture mixture (500 ml) was obtained by shaking the culture after 3 days in King’s medium B (KB: proteose peptone No.3 20 g; glycerin 10 ml; K_2_HPO_4_⋅3H_2_O 1.5 g; MgSO_4_⋅7H_2_O 1.5 g; agar 18 g; distilled water 1000 ml; and pH 7.0) with 160 rpm at 28°C, and it was adjusted to an optical density of OD_600_ = 0.5 using a spectrophotometer (GE Uitrospec 2100 pro).

### Field Experiment of *Panax notoginseng* and Soil Collection

The field experiment was conducted in July, 2017 at a *P. notoginseng* commercial farm located in Hexi Town, Tonghai County of Yunnan Province, China (102.75° = E, 24.12° = N, 1899.8 m Alt.), and field experiment layout (Supplementary Figure S1) samples were collected in September 2017 from different plots in the same field with arid continental climate and laterite soil. *P. notoginseng* was cultivated strictly according to standard operating procedures established by the Good Agriculture Practices (Heuberger et al., 2010) and was consecutively grown for 3 years. A ridge cultivation pattern was used, with ridges approximately (1.3 m × 15 m). The experiment was conducted as a block design with three replicates; three plots per treatment served as replicates, the area of each replicate plot was (1.3 m × 1.5 m), and the root-rot plants plot and death plants plot (as control 1 and control 2, respectively) were (1.3 m × 3 m) under the same management. *P. notoginseng* was cultivated in the soil for the first time, and, after the cultivation of *P. notoginseng*, no other crop was grown. Each treatment has about 145–170 plants of *P. notoginseng*, and the probiotic consortia root was irrigated one time for every 7 days. The treatment was continuous for three times, and each plant was irrigated with 150 ml probiotic consortia culture mixture (5 × 10^6^ CFU/ml).

A collection of *P. notoginseng* rhizosphere soil was made, and samples were taken from a depth of 20 cm using a shovel. In this experiment, four treatment groups (A, B, C, and D) and two control groups (JKT and BT) were designed. Treatments A, B, C, and D represent four probiotic consortia, probiotic consortia A (three strains of *Lysobacter* communities), probiotic consortia B (one strain of *Lysobacter*, one strain of *Pseudomonas*, and two strains of *Bacillus* communities), probiotic consortia C (four strains of *Bacillus* communities) and probiotic consortia D (eight strains of *Bacillus* communities). E represents the biopesticide (Shandong Kaoshan Biotechnology Co., Ltd., China), and JKT represents light diseased *P. notoginseng* (control) treated with water. BT represents severe diseased *P. notoginseng* (control) treated with water. We collected rhizosphere soil of groups of A, B, C, D, E, JKT, and BT (a region of the soil, about 1 mm surrounding roots) (Lundberg et al., 2012). Samples of *P. notoginseng* rhizosphere soil were placed in 50 ml sterile centrifuge tubes, stored at −80°C Ultra-low temperature freezer immediately, and then sealed in a dry ice box and sent to OE Biotech Co., Ltd. (Shanghai, China).

### Evaluation of Control Efficiency and the Quality of *Panax notoginseng*

We applied three applications (7 days interval) of probiotic consortia in July, and a survey was conducted at harvesting time at the end of November to evaluate the death rate of *P. notoginseng* plants from each treatment and the total number of plants from each plot. The death rate was calculated for each plot as the number of dead *P. notoginseng* plants divided by the total number of *P. notoginseng* plants in each plot. The mean value of the fresh weight, dry weight, and root length of *P. notoginseng* were calculated in each treatment.

We assessed the ginsenoside contents of five major saponins (R1, Rg1, Re, Rb1, and Rd) in the crude ginseng saponin fraction from *P. notoginseng* root powder for each treatment (Yang et al., 2015). We used standard curves to quantify the concentration of ginsenoside in samples that the linear relationships between the peak areas and the concentrations. The ginsenoside contents of the samples were calculated by using the following formula:

$$\mathrm{Content}\,\mathrm{of}\,\mathrm{ginsenoside}\,\mathrm{in}\,\mathrm{roots}\,\mathrm{or}\,\mathrm{soil}\,\left(\mu \,g/g\right)=1000\times \left(m\times V\right)/W{}_{1}$$

where “m” is the ginsenoside content of sample extract as determined from a standard curve (mg/ml); “V” is the methanol volume (ml) to dissolve the dry residue of sample extract; and “W_1_” is the weight of the sample (g).

### DNA Extraction and Library Construction

Extraction of DNA was done from 0.5 g of soil; for DNA extraction, we used a Power Soil^®^ DNA isolation kit (MO BIO Laboratories, Inc., Carlsbad, CA, United States) following the manufacturer’s instructions, and extracted DNA was stored at −80°C for future use. Bacterial diversity was analyzed by using V3–V4 variable regions of 16S rRNA genes, and these were amplified with universal primers 343F (5′-TACGGRAGGCAGCAG-3′) and 798R (5′-AGGGTATCTAATCCT-3′). Fungal diversity was analyzed by using ITS1 variable regions, and these were amplified with universal primers 1743F (5′-CTTGGTCATTTAGAGGAAGTAA-3′) and 2043R (5′-GCTGC GTTCTTCATCGATGC-3′) (Zhu et al., 2018).

By using gel electrophoresis, amplicon quality was visualized, and purification was done with the help of AMPure XP beads (Agencourt); PCR (Bio-rad, Cat. No. 580BR10905) was done for amplicon. AMPure XP beads were run again for the purification of amplicon, and quantification of the final amplicon was done by using Qubit dsDNA assay kit (Life Technologies Cat. No. Q328520). The same numbers of purified amplicons were pooled for subsequent sequencing (Gao et al., 2018).

### Bioinformatics Analysis

For DNA sequencing, raw data was collected in the FASTQ format. The ambiguous base (N) was detected and deleted by using the Trimmomatic software preprocessing the paired-end reads (Bolger et al., 2014). It also cut off low-quality sequences with an average quality score below 20 by sliding window trimming approach. After trimming, paired-end reads were assembled using FLASH software (Reyon et al., 2012). Parameters of the assembly were 10 bp of minimal overlapping, 200 bp of maximum overlapping, and 20% of maximum mismatch rate. Sequences were performed for further denoising: reads with ambiguous, homologous sequences or below 200 bp were abandoned. Reads with 75% of bases above Q20 were retained. Then, reads with chimera were detected and removed. These two steps were achieved using QIIME software (Caporaso et al., 2010).

Clean reads were subjected to primer sequence removal and clustering to generate operational taxonomic units (OTUs) using UPARSE software with a 97% similarity cutoff (Edgar, 2013). The representative read of each bacterial OTUs was selected using the QIIME package. All representative reads were annotated and blasted against Silva database (Greengenes) (16S rDNA) using the RDP classifier for bacteria (confidence threshold was 70%) (Wang et al., 2007; Chen et al., 2019). The representative reads of each fungal OTUs were selected using the QIIME package. All representative reads were annotated and blasted against the unite database for fungi using blast (Altschul et al., 1990). All sequences of ITS and 16S rRNA genes can be found in the Short Read Archive (SAR) at NCBI under accession number PRJNA588201.

OTUs tables were used to calculate different alpha diversity metrics, including observed OTUs, Chao 1, Shannon, and Simpson indices using the QIIME. To understand variation in the bacterial and fungal community structure across the treatments, weighted UniFrac and unweighted UniFrac distances were performed in QIIME. The relative abundance bar plots and heatmap for both bacterial and fungal genera, and distance heatmaps for seven soil samples were generated using R scripts executed in Rv3.5.3. Analysis of variance (ANOVA) was performed, showing the least significant difference (LSD) was 5% for these variables in all treatment replicated to analyze the death rates. Data were analyzed statistically using ANOVA (*P* < 0.05). The means were compared using Tukey’s test (*P* < 0.05) (Dong et al., 2016).

## Results

### Evaluation of the Growth Characteristics, Death Rate, and Quality of *Panax notoginseng*

The effect of probiotic consortia on *P. notoginseng* roots and its potential beneficial impact on the host plants have been assessed. The root microbiota is also beneficial to the growth and health of host plants (Lee and Park, 2004). We compared the death rate, fresh root weight, dry root weight, and root length of the probiotic consortia treated plants. Compared to the control BT, the death rate of *P. notoginseng* was significantly reduced by probiotic consortia (A, B, and D) and the biopesticide (E) (*p* < 0.05), and the mortality rate was decreased by 22.11, 24.12, and 33.09%, respectively (Supplementary Table S3). The fresh root weight increased significantly by use of probiotic consortia (A and B) and the bio-pesticide (E) (*p* < 0.05); they weighed 19.33, 17.47, and 17.77, respectively. The weight of BT in the control group was 10.50 g. However, the parameters of root length and root dry weight exhibited no significant differences. These data indicated that probiotic consortia as a potent inhibitor was present in the rhizosphere soil of *P. notoginseng*, contributed to increase root weight and reducing the death rate of root-rot disease (Supplementary Table S4).

**FIGURE 1:**
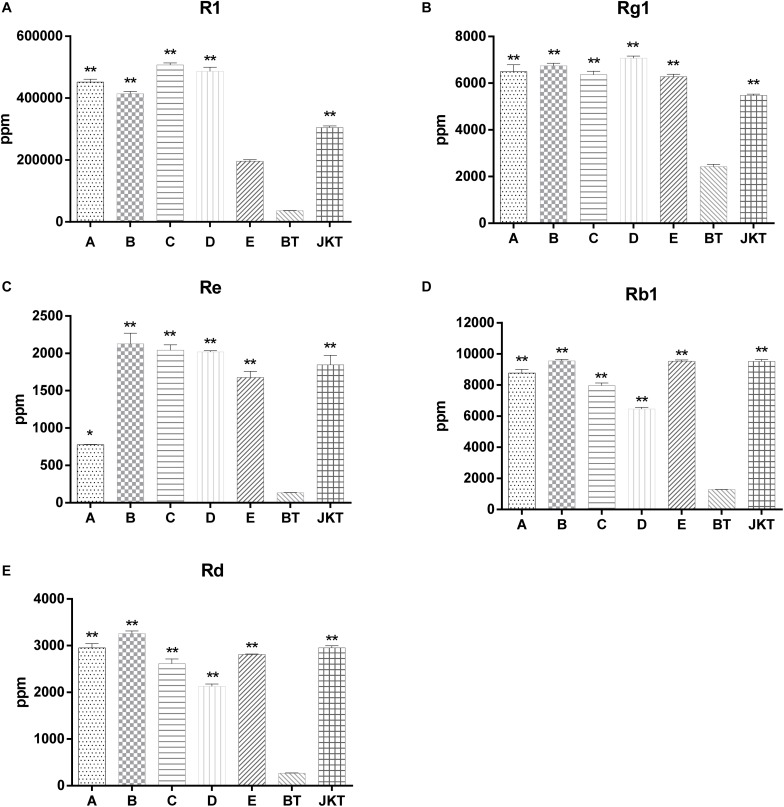
The significant difference between five major saponins (R1, Rg1, Re, Rb1, and Rd) in *P. notoginseng* compared to severe diseased plants (BT). **A**, **B**, **C**, and **D** represents four probiotic consortia; **E** represents biopesticide, JKT; and BT represents healthy plants and diseased plants without any treatment. LSD test was used and at a *p*-value <0.05 marked as * and a *p*-value <0.01 marked as **.

Besides these, five major saponins (R1, Rg1, Re, Rb1, and Rd) in *P. notoginseng* roots were determined by HPLC, and the results showed explicitly that five major saponins were the significant difference between four probiotic consortia treatments and the control (diseased plants) (Figure 1). Probiotic consortia (B) treatment increased the three saponin contents the most (Re, Rb1, and Rd). Probiotic consortia (C) treatment increased saponins R1 the most. Probiotic consortia (D) treatment increased saponins Rg1 the most (Supplementary Table S5). These results revealed that the successive inoculation of probiotic consortia affected the assembly of bacterial and fungal communities in the rhizosphere for disease suppression.

### Effect of Probiotic Consortia on Diversity and Structure of Microbial Community

**FIGURE 2:**
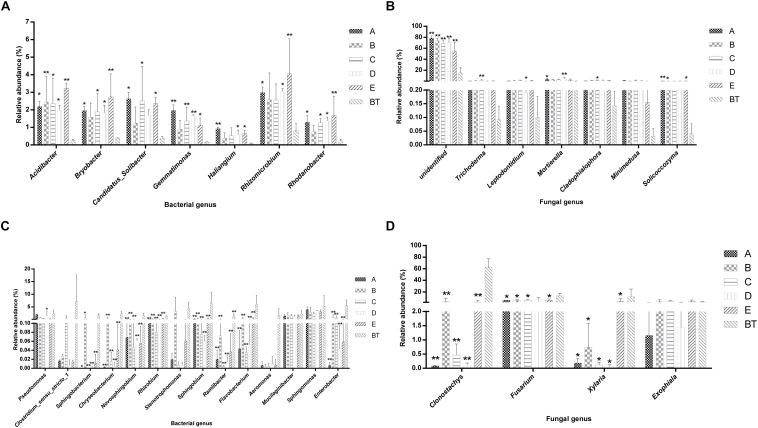
Column chart analysis of dominant bacterial and fungal genera in rhizospheric soil of plants. **(A)** Increase of relative abundance of potential beneficial bacterial genera. **(B)** Increase of relative abundance of potential beneficial fungal genera. **(C)** Decrease of relative abundance of potential harmful bacterial genera. **(D)** Decrease of relative abundance of potential harmful fungal genera.

The species richness (OTUs, 97%) and species diversity (Chao1, Shannon, and Simpson index, 97%) are shown in Supplementary Table S6. In all treatments, richness and diversity exhibited no significant difference when compared to control JKT. The Simpson index showed that there was no significant difference for bacterial species diversity in all soil samples, while (Chao1, Shannon) index expressed that species richness among five treatments (A, B, C, D, and E), and species diversity among four treatments (A, C, D, and E) showed a significant difference compared to control BT. Bacterial richness and diversity indices were highest in all five treatment soil samples (Supplementary Table S6). We found that, after inoculation with probiotic consortia, the treatment groups (A, B, C, and D) were higher in bacterial and fungal OTUs than the control BT (Supplementary Figure S2). Microbial diversity of most of the bacterial and fungal genera was found to be a difference in rhizosphere soil of different treatments as compared to control. Compared to BT, four probiotic consortia treatments and one biopesticide treatment all increased the relative abundance of seven dominant bacterial genera (Figure 2A) and seven dominant fungal genera (Figure 2B) in rhizosphere soil of diseased plants. Moreover, four probiotic consortia treatments and one biopesticide treatment all decreased the relative abundance of 14 dominant bacterial genera (Figure 2C) and four dominant fungal genera (Figure 2D) in rhizosphere soil.

**FIGURE 3:**
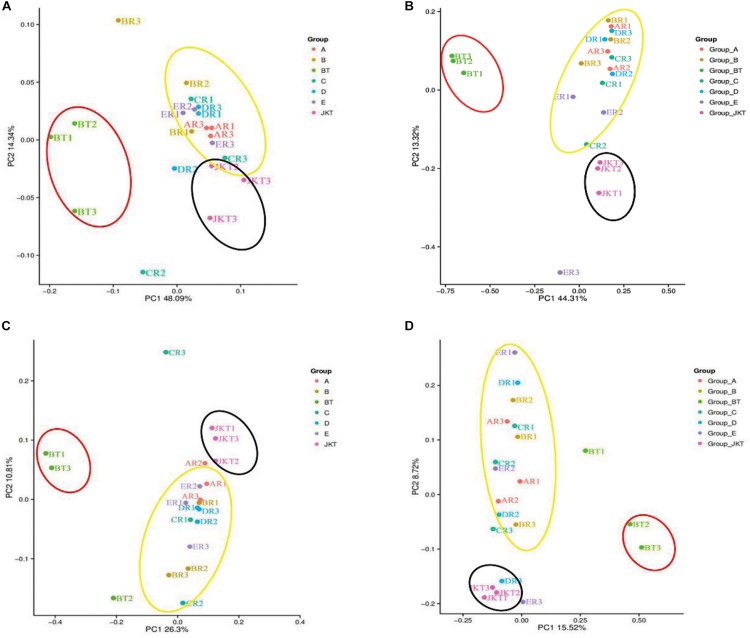
Microbial community structures in the seven different soil samples. Weighted UniFrac principal coordinate analysis of bacterial **(A)** and fungal **(B)** community structures, and unweighted UniFrac principal coordinate analysis of bacterial **(C)** and fungal **(D)** community structures. Group A, B, C, and D represent four probiotic consortia, Group E represents the biopesticide, and Group BT and Group JKT represents the controls without any treatment.

**FIGURE 4:**
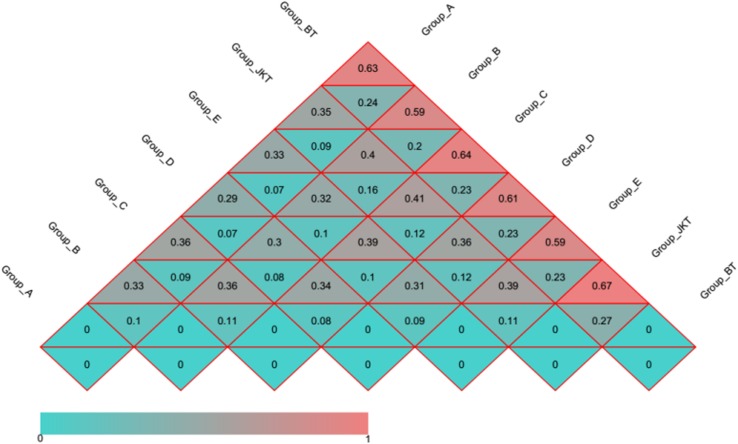
Distance heatmap graph of seven soil samples. Weighted UniFrac and unweighted UniFrac analysis. The number in the grid is the coefficient of dissimilarity between two samples. The smaller the coefficient of dissimilarity, the smaller the diversity of species. In the same grid, the upper and lower values represent the distance between unweighted UniFrac and weighted UniFrac, respectively.

Weighted UniFrac (based on abundances of taxa) and unweighted UniFrac (sensitive to rare taxa) were used to distance metrics to estimate β-diversity. With the unconstrained principal coordinate analysis (PCoA) of weighted UniFrac and unweighted UniFrac distances showing that the majority of the variation in microbial diversity across the four probiotic consortia treatments and two controls could be attributed to the field (Figure 3). Both weighted UniFrac and unweighted UniFrac distances revealed that the control BT (weighted UniFrac *R*^2^ ≥ 0.20, unweighted UniFrac *R*^2^ ≥ 0.59) were separated from the other soil samples along with the first component (PC1) for both bacterial and fungal communities, and the treatment soil samples and control JKT were in the same direction (Figure 4). After treatment by four probiotic consortia treatments (A, B, C, and D) and the biopesticide (E), the results indicated that the microbe structure of the rhizosphere soil of plants treated with probiotic consortia was similar to healthy plants and differed from themicrobe structure of diseased plants.

### Effect of Probiotic Consortia on Bacterial Community Composition

**FIGURE 5:**
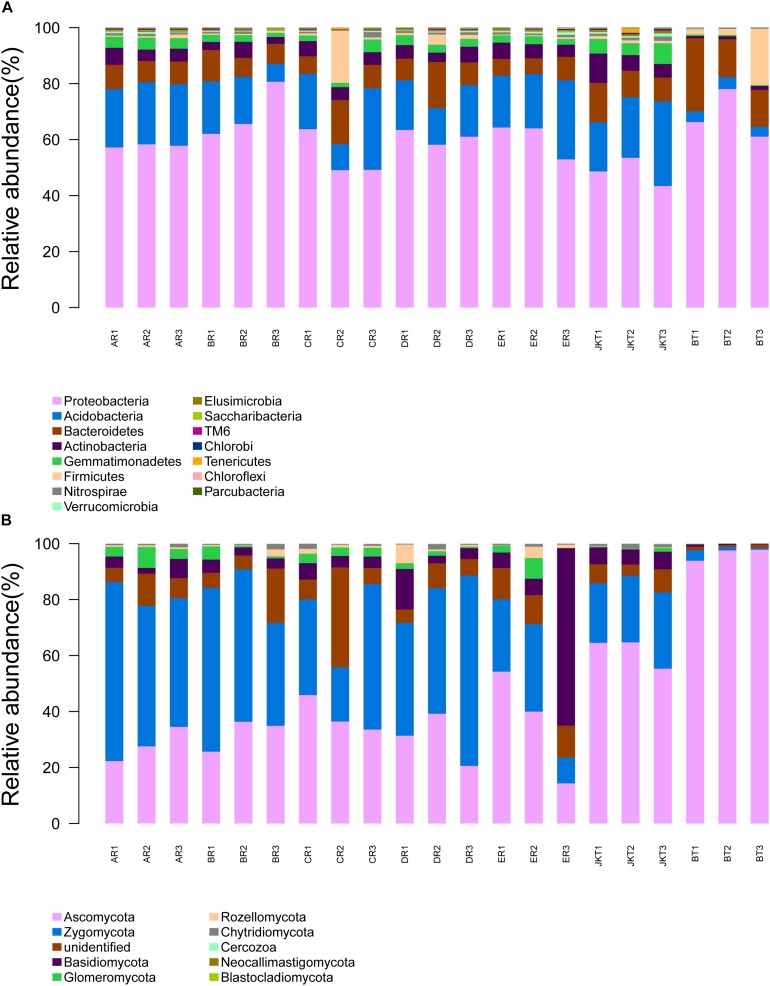
Relative abundance at the phylum level. **(A)** Relative abundance at the phylum level in bacterial communities. **(B)** Relative abundance at the phylum level in fungal communities. Based on the species annotation results, we chose to examine the distribution of relative species abundance among the top-ranked phyla; the relative abundance distribution of the top 15 species **(A)** and the top 10 species **(B)** are shown. Each soil sample all collected from three replicate plots.

The dominant bacterial phyla in all rhizospheric soil were Proteobacteria, Acidobacteria, and Bacteroidetes (average abundance was 59.61, 17.11, and 10.29% of all samples, respectively), followed by Actinobacteria (4.42%), Gemmatimonadetes (2.84%), Firmicutes (2.38%), and Nitrospirae (0.16%) (Figure 5A). Relative abundance at the phylum level in bacterial communities of the top 15 species revealed significant differences among groups (Supplementary Table S7). Other phyla, such as Verrucomicrobia, Elusimicrobia, Saccharibacteria, TM6, Chlorobi, Tenericutes, Chloroflexi, and Parcubacteria, were found at <1% in relative abundance in all of the samples. Proteobacteria was the most dominant phylum in all soil samples and had a minimum frequency (48.03%). The proportion of Actinobacteria was the lowest in the control of BT soil samples (1.09%). Proteobacteria was the most abundant phylum in the B, D, E, and BT soil samples, and Acidobacteria was the most abundant phylum in the A, E, and JKT soil samples. The proportion of Proteobacteria was the highest in the control of BT soil samples (68.34%) (Supplementary Table S8).

Relative abundance analysis at the genus level showed that healthy and diseased plants affected the microbial community composition in rhizospheric soil. By classifying all OTUs into taxonomic groups, we identified 27 bacterial genera (Difference value of relative abundance >1.00%, Supplementary Table S9) associated with rhizospheric soil of plants with light and over-serious disease. The dominant bacterial genera in light diseased plants (JKT) were *Acidibacter*, *Bryobacter*, *Candidatus Solibacter*, *Gemmatimonas*, etc. The relative abundance was 2.34, 2.42, 3.20, and 3.00%, respectively. However, in the BT control group, the richness was only occupied 0.25, 0.38, 0.39, and 0.15%, respectively. However, *Chryseobacterium*, *Enterobacter*, *Flavobacterium*, and *Pseudomonas*, etc. were found to be the dominant bacterial genera in rhizospheric soil of plants with the over-serious disease (BT), and the relative abundance was 2.25, 5.61, 5.96, and 2.30%, respectively. It was completely different from light diseased plants (JKT), where the richness was 0.01, 0.00, 0.05, and 0.09%, repectively.

### Effect of Probiotic Consortia on Fungal Community Composition

The dominant fungal phyla in all rhizospheric soil were Ascomycota, Zygomycota, Unidentified, and Basidiomycota (average relative abundance 46.23, 33.94, 8.43, and 7.22% of all samples, respectively), followed by Glomeromycota (2.10%) and Rozellomycota (1.12%) (Figure 5B). Relative abundance at the phylum level in fungal communities of the top 10 species revealed significant differences among groups (Supplementary Table S10). Other phyla, such as Chytridiomycota, Cercozoa, Neocallimastigomycota, and Blastocladiomycota, were found at <1% in relative abundance in all samples. Ascomycota was the most abundant phylum in the C, E, JKT, and BT soil samples, and Zygomycota was the most abundant phylum in the A, B, and D soil samples. The proportion of Ascomycota was the highest in the control of BT soil samples (96.40%) (Supplementary Table S8).

Relative abundance analysis at the genus level showed that healthy and diseased plants affected the microbial community composition in rhizospheric soil. By classifying all OTUs into taxonomic groups, we identified 11 fungal genera (Difference value of relative abundance >1.00%, Supplementary Table S11) associated with rhizospheric soil of plants with light and over-serious disease. The dominant fungal genera in rhizospheric soil of plants with the light disease (JKT) were *Leptodontidium*, *Mortierella*, and *Trichoderma*, etc. The relative abundance was 3.67, 2.46, and 5.49%, repsectively; and richness was 0.10, 0.30, and 0.09%, respectively, thus being more abundant than in diseased plants (BT). *Clonostachys*, *Exophiala*, *Fusarium*, and *Xylaria* were, however, the more abundant genera in the rhizospheric soil of plants with the severe disease. These four genera accounted for 0.12, 0.47, 2.35, and 0.80% of the control JKT, respectively. However, they also accounted for 62.73, 12.42, 13.11, and 11.47, respectively, in control BT.

### Microbial Community Composition and Differentially Abundant Taxa

At the genus level, the 30 most abundant genera were selected to construct the bacterial genera abundance map and the fungal genera abundance map based on the clustering analysis of genera species abundance differences, revealing the differences in bacterial and fungal diversity (Supplementary Figures S3a,b). Based on the bacterial genera present in the samples (Supplementary Figure S3a), a cluster containing two or three duplicate soil samples were considered to be clustered into one category. All soil samples were clustered into four groups. Control (BT) soil samples notably clustered into one group, while soil samples (B) and soil samples (D) were clustered into another group. Soil samples (E) clustered together, and soil samples (A) and control (JKT) soil samples clustered together roughly.

Further, relatively high proportions of bacterial genera, such as *Sphingobacterium*, *Flavobacterium*, *Chryseobacterium*, and *Enterobacter*, were found among the rhizospheric soil of death plant (control BT) affected by root-rot disease. Based on the fungal genera present in the samples (Supplementary Figure S3b), a cluster containing two or three duplicate soil samples wereconsidered to be clustered into one category. So, all samples notably clustered into two groups. Control (BT) soil samples notably clustered into one category, while other soil samples were clustered into another group. Additionally, relatively high proportions of fungal genera such as *Clonostachys*, *Fusarium*, *Xylaria*, and *Monographella* were found among the rhizospheric soil of root-rot disease plants.

## Discussion

*Panax notoginseng* herbaceous perennial plants that grow in specialized surroundings are primarily cultivated in artificial shade for several years. For consecutive cultivation of *P. notoginseng*, seedling replanting is a significant issue, which is made more difficult by multiple factors, such as soil-borne pathogens accumulated in rhizosphere soil resulting in *P. notoginseng* root-rot diseases (Mao et al., 2014). So, we need to find a solution to root-rot by inoculating probiotics into the roots of diseased plants. Here, we have studied the probiotic bacterial community performance and its potential beneficial effects in microbial diversity, and there is a precedent for the use of microbial communities to study plant–microbe interactions and microbe–microbe interactions (Santhanam et al., 2015).

### Quality of *P. notoginseng* Improved with the Inoculation of Probiotic Consortia

Microbial diversity is critical to maintaining soil health and quality, and it serves as a sensitive bioindicator of soil health as well. For example, microbial diversity and root disease suppression are related. In recent years, there have been more and more studies conducted on the control of root-rot of ginseng with microbial antagonists, but there are few studies on root-rot of *P. notoginseng* (Ryu et al., 2014; Song et al., 2014). Therefore, in the present study, we have assembled the probiotic consortia and examined the mechanisms linking probiotic consortia with suppression of root-rot disease. We screened four probiotic consortia by using 13 antagonistic strains, and the 3-year-old *P. notoginseng* field, which fell pray to serious root-rot disease, was used as the primary research object. We observed that the two probiotic consortia (A, B) treatments significantly increased the fresh root weight, and three probiotic consortia treatments (A, B, and D) significantly reduced the root-rot disease death rate compared to the control (BT). However, no significant difference in death rate between probiotic consortia C and control (BT) was observed. Probiotic consortia B treatment mostly increases the three saponin contents (Re, Rb1, and Rd), probiotic consortia C treatment mostly increased saponins R1, and probiotic consortia D treatment mostly increased saponins Rg1 compared to the BT control. Moreover, four probiotic consortia treatments significantly enhanced the major saponin content compared to the BT control. Thus, applying the probiotic consortia could not only be used for biological control of plant disease but also the improvement of plant growth and quality of *P. notoginseng* (Xia et al., 2017). The application of probiotic consortia changes the imbalance of micro-ecology to the state of relative health and reducing plant mortality.

### Inoculation of Probiotic Consortia May Indirectly Promote the Growth of Other Beneficial Bacteria

Even though inoculated probiotic consortia B and probiotic consortia C were not normally or significantly increased. *Pseudomonas*, *Bacillus* spp., and *Lysobacter* spp. levels may have increased other beneficial biocontrol bacteria indirectly just as *Pseudomonas* spp. was increased by probiotic consortia A and probiotic consortia D. The results of this study showed that the introduced the microbes (*Bacillus*, *Lysobacter*, and *Pseudomonas*) have a specific endurance capability and could improve the nutritional status of plant rhizosphere; their abundance has a significant effect on the diversity of *F. oxysporum* and also for the suppression of root-rot disease (Qian et al., 2009; Verbon and Liberman, 2016). The three probiotic consortia (A, B, and C) treatments significantly decreased the abundance of *F. oxysporum* as compared to control (BT), signifying that probiotic consortia were able to develop a soil resistance against *Fusarium* root-rot disease. *F. oxysporum*, which causes the *Fusarium* wilt of vanilla, was significantly reduced by the application of biofertilizer (Xiong et al., 2017). Inoculation of probiotic consortia may indirectly promote the growth of other beneficial bacteria and fungi, thereby suppressing root-rot. We found after sequencing that the abundance of *Trichoderma* in treatments A, B, C, and D gave better results as compared with control BT group; *Trichoderma* has been reported to be a biocontrol fungus having a wide range of antagonistic properties, and it can effectively inhibit the growth of *F. oxysporum* and *Fusarium solani* (Luo et al., 2019; Sallam et al., 2019). There was a negative relationship between the probiotic consortia abundance and *Fusarium* abundance, and inoculated probiotic consortia constrained *Fusarium* pathogen density. We therefore cannot ignore the role of probiotics to control plant diseases.

### Soil Microbial Community Structure Changes With the Inoculation of Probiotic Consortia

After the application of probiotic consortia, unusual changes were observed in the microbial community, and PCoA results showed that all four probiotic consortia harbored distinct microbial communities structurally. The rhizosphere soil was treated by the different probiotic consortia and exhibited different microbial communities, and these changes were associated with observed patterns of *Fusarium* pathogen abundance and the incidence of root-rot disease. The significant differences of the bacterial community were found in the rhizosphere soil of susceptible disease (control JKT) and dead *P. notoginseng* (control BT); lower bacterial diversity was found in the rhizospheric soil of dead *P. notoginseng* as compared to susceptible diseased plants. The previous studies have supported this view that continuous *ginseng* planting decreased the bacteria diversity and aggravated root-rot disease (Mendes et al., 2011; Santhanam et al., 2015). The application of four probiotic consortia was a significant factor in reshaping the soil community taxonomic composition, which was the same as that observed in the application of two biofertilizers previously. Because the limited read lengths of the Illumina MiSeq sequencing do not allow for robust taxonomic characterization to the species level, we focused our examination of microbial composition changes at the genus level. *Acidibacter*, *Candidatus*, *Solibacter*, *Rhizomicrobium*, and *Gemmatimonas* were over-represented in the four probiotic consortia treatments. Some taxa of the acido-bacteria have been found previously to be more abundant in potato common scab disease-suppressive soil as compared to conducive soil (Rosenzweig et al., 2012). However, the abundance of *Flavobacterium*, *Clostridium*, *Chryseobacterium*, and *Enterobacter* genera was decreased. In our study, we were tested about 10 strains isolated from diseased *P. notoginseng* plants, which belonged to *Chryseobacterium*, and *Enterobacter* genera, which caused *P. notoginseng* plants death (unpublished data), suggested these genera were positively correlated with plant death of *P. notoginseng*. Furthermore, the genera of *Aeromonas*, *Chryseobacterium*, *Clostridium sensu stricto 1*, *Flavobacterium*, *Enterobacter*, and *Sphingobium* showed massive accumulation in the rhizosphere soil of dead plants (control BT); four treatments, which are made of potential pathogenic bacteria, cause aquaculture animals disease and human and animal diseases (Wahli et al., 2005; Zhu et al., 2010). So, these genera may be important bacterial pathogens that positively contribute to the effects of *P. notoginseng* plant death.

The microbial diversity and structure in the rhizosphere of *P. notoginseng* and seedling growth, relieving the occurrence of root-rot diseases, were affected by the application of probiotic consortia. Our study provides strong evidence in support of the hypothesis that inoculated probiotic consortia ameliorated rhizosphere microflora to restrict disease outbreaks during continuous *P. notoginseng* cropping. Due to the high complexity of soil microorganisms, studies on the mechanism of soil microbial community to protect plants from pathogen infection are still limited. Therefore, further studies will need to identify the role of beneficial bacteria and construct more groups of probiotic consortia that can be applied in the rhizosphere against root-rot of *P. notoginseng*. The role of probiotic consortia in regulating the mechanism of *P. notoginseng* root-rot still needs further exploration.

## Conclusion

After this study, we came to the conclusion that probiotic consortia in the form of different beneficial bacterial strains positively affected the bacterial and fungal communities to display some kind of disease suppressive through reshaping the function and structure of soil microbes. By introducing the probiotic consortia could have a possible role in the direct or indirect alleviation of *P. notoginseng* root-rot in the continuous cropping soil. Moreover, additional groups of probiotic consortia that showed positive synergism in the soil rhizosphere against different diseases including root-rot disease must be considered. So, the disease suppressive properties of such kinds of probiotic consortia to control the devastating diseases of plants should not be ignored in the future.

## Data Availability Statement

The datasets generated for this study can be found in the All sequences of ITS and 16S rRNA genes can be found in the Short Read Archive (SAR) at NCBI under accession number PRJNA588201.

## Author Contributions

GJ and LF conceived and designed the experiments. JZ, LW, JY, and YW performed the experiments. JZ and LW analyzed the data. GJ and LF contributed reagents, materials, and analysis tools. JZ, LF, and WA wrote the manuscript.

## Conflict of Interest

The authors declare that the research was conducted in the absence of any commercial or financial relationships that could be construed as a potential conflict of interest.

## Supplementary Material

FIGURE S1 Field experiment layout. A, B, C, and D represents four probiotics consortia, E represents biopesticide (Shandong Kaoshan Biotechnology Co., Ltd., China), and JKT represents light diseased plants treated with water. BT represents severe diseased plants (control) treated with water. R and T represent repeats.

FIGURE S2 Comparison of Bacterial **(a)** and Fungal **(b)** OTUs control group JKT and BT in treatment group A, B, C, D, and E. A, B, C, and D represents four probiotics consortia, E represents biopesticide (Shandong Kaoshan Biotechnology Co., Ltd., China), and JKT represents light diseased plants treated with water. BT represents severe diseased plants (control) treated with water.

FIGURE S3 Heatmap representing their relative abundance. **(a)**. Heatmap analysis showing the distribution of the core bacterial genera. **(b)**. Heatmap analysis showing the distribution of the core fungal genera. Generation of a mapping image based on abundance in formation. To examine the abundance information available at the species level, we selected genera represented by the 30 most abundant sequences to draw a species abundance cluster map. Abscissae indicate sample information, and ordinates indicate species annotation information. The cluster tree on the left side of the figure is a species cluster tree; a sample cluster tree is shown above the cluster tree. Each soil sample all collected from three replicate plots.

TABLE S1 List of the bacteria strains used in this study.

TABLE S2 Four probiotic consortia assembled by 13 different bacteria strains.

TABLE S3 Suppression effects on diseased *Panax notoginseng*.

TABLE S4 Inhibition effects of the probiotic consortia against root-rot disease and quality evaluation of *Panax notoginseng*.

TABLE S5 Five major saponins (R1, Rg1, Re, Rb1, and Rd) in *P. notoginseng*.

TABLE S6 Diversity indices of bacterial and fungal communities in rhizospheric soil.

TABLE S7 Relative abundance at the phylum level in bacterial communities.

TABLE S8 Classification according to the taxonomic units of phylum (abundance > 1%).

TABLE S9 Comparison and analysis of the bacteria genera in rhizospheric soil of light diseased plants and severe diseased plants.

TABLE S10 Relative abundance at the phylum level in fungal communities.

TABLE S11 Comparison and analysis of the fungal genera in rhizospheric soil of lightly diseased plants and severely diseased plants.
