# Supplementary figures and images for: Probiotic Consortia: Reshaping the Rhizospheric Microbiome and Its Role in Suppressing Root-Rot Disease of Panax notoginseng
Source: Front Microbiol. 2020 Apr 30;11:701. doi: 10.3389/fmicb.2020.00701 (PMC7203884; doi:10.3389/fmicb.2020.00701)

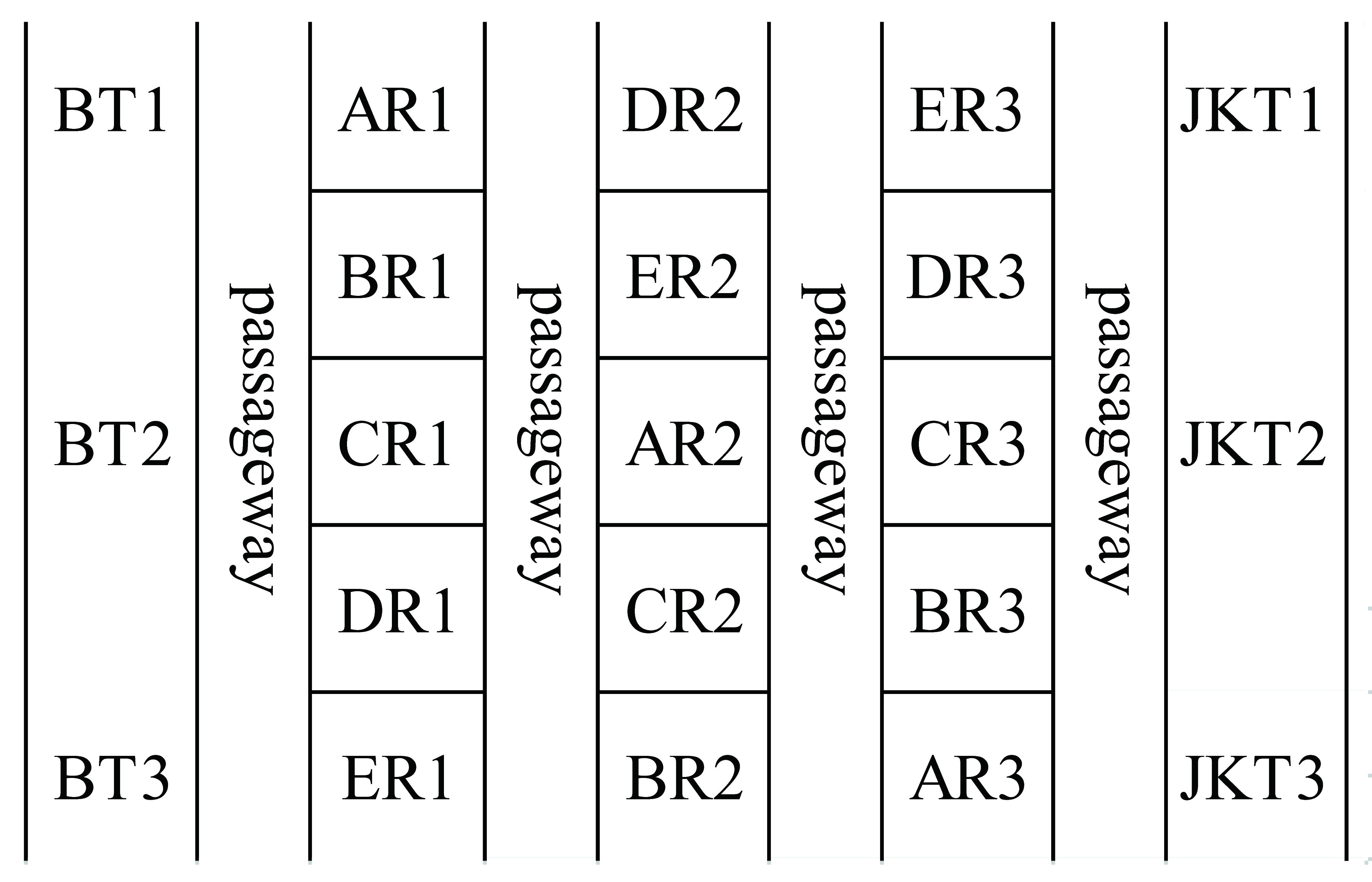

Supplement: FIGURE S1 — Field experiment layout. A, B, C, and D represents four probiotics consortia, E represents biopesticide (Shandong Kaoshan Biotechnology Co., Ltd., China), and JKT represents light diseased plants treated with water. BT represents severe diseased plants (control) treated with water. R and T represent repeats. [file Image_1.TIF]

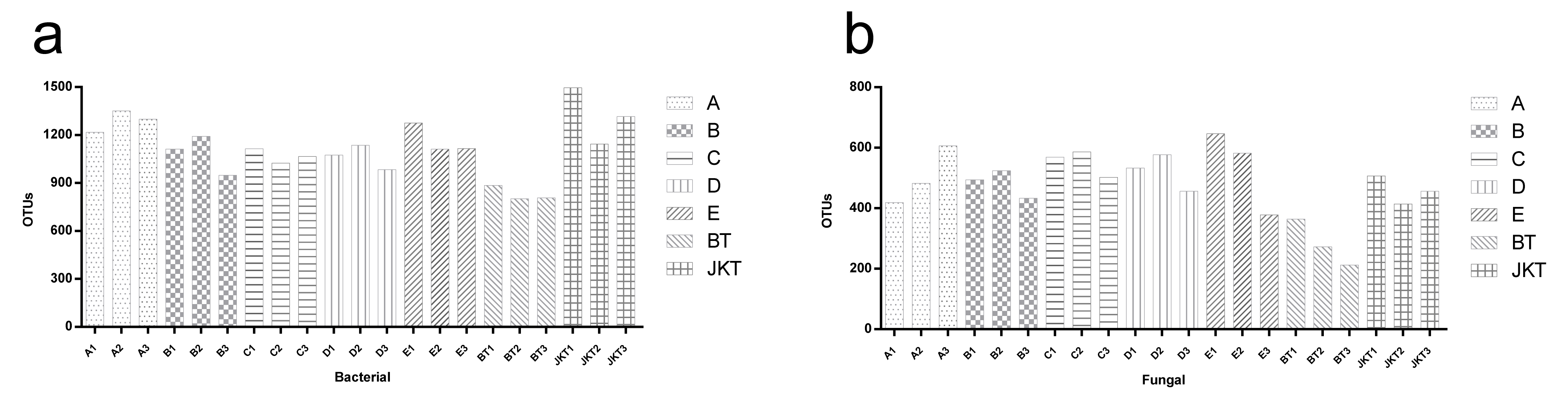

Supplement: FIGURE S2 — Comparison of Bacterial (a) and Fungal (b) OTUs control group JKT and BT in treatment group A, B, C, D, and E. A, B, C, and D represents four probiotics consortia, E represents biopesticide (Shandong Kaoshan Biotechnology Co., Ltd., China), and JKT represents light diseased plants treated with water. BT represents severe diseased plants (control) treated with water. [file Image_2.tif]

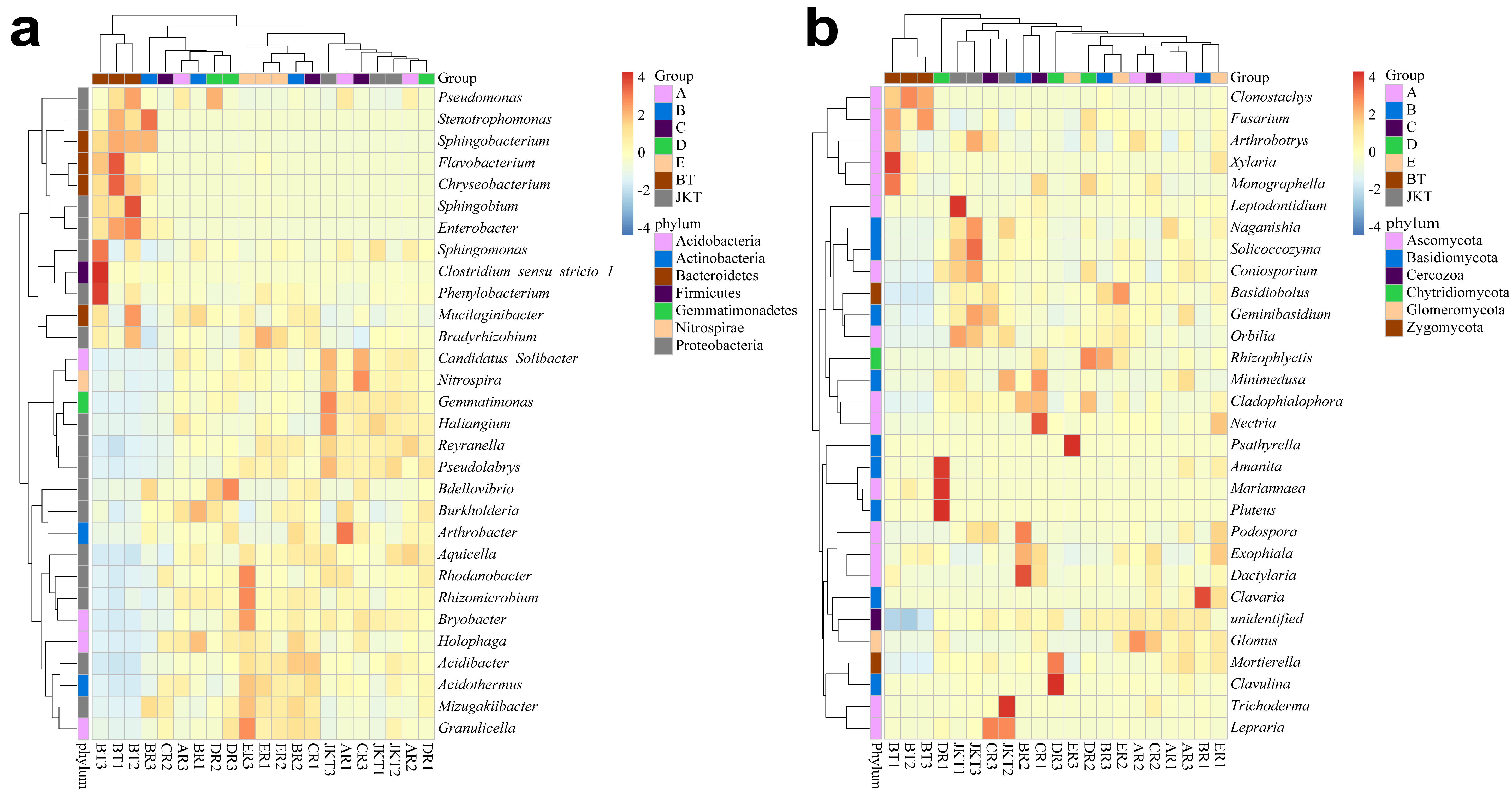

Supplement: FIGURE S3 — Heatmap representing their relative abundance. (a). Heatmap analysis showing the distribution of the core bacterial genera. (b). Heatmap analysis showing the distribution of the core fungal genera. Generation of a mapping image based on abundance in formation. To examine the abundance information available at the species level, we selected genera represented by the 30 most abundant sequences to draw a species abundance cluster map. Abscissae indicate sample information, and ordinates indicate species annotation information. The cluster tree on the left side of the figure is a species cluster tree; a sample cluster tree is shown above the cluster tree. Each soil sample all collected from three replicate plots. [file Image_3.TIF]
