# Supplementary material for: Probiotic Consortia: Reshaping the Rhizospheric Microbiome and Its Role in Suppressing Root-Rot Disease of Panax notoginseng
Source: Front Microbiol. 2020 Apr 30;11:701. doi: 10.3389/fmicb.2020.00701 (PMC7203884; doi:10.3389/fmicb.2020.00701)
Supplement: TABLE S1 — List of the bacteria strains used in this study. [file Table_1.DOCX]

**Table S1.** **List of the bacteria strains used in this study**

| Species | GenBank No. | Origin |
| --- | --- | --- |
| *Bacillus amyloliquefaciens* C3 | HQ668178.1 | Rhizosphere Soil of Amorphophallus konjac from Fuyuan County of Yunnan Province, PRC |
| *Bacillus methylotrophicus* R2-2 | JN648098.1 | Rhizosphere Soil of Amorphophallus konjac from Fuyuan County of Yunnan Province, PRC |
| *Bacillus amyloliquefaciens* 2-B-39 | MH028958 | Root tissue of *Panax Notoginseng* from Tonghai County of Yunnan Province, PRC |
| *Bacillus velezensis* YQ-11 | KY427070.1 | Rhizosphere Soil of pine tree from Sedan - chair snow mountain of Yunnan Province, PRC |
| *Bacillus velezensis* SQ-5 | KY427069.1 | Rhizosphere Soil of *Panax Notoginseng* from Funing County of Yunnan Province, PRC |
| *Bacillus subtilis* B908 | MH028960 | Soils of Beijing suburbs, PRC, strain for commercial bio -product |
| *Bacillus subtilis* M3 | HQ825324.1 | Endophytic bacteria of Amorphophallus konjac in Fuyuan County of Yunnan Province, PRC |
| *Bacillus subtilis* 1-JKT-10 | MH028957 | Root tissue of *Panax Notoginseng* from Tonghai County of Yunnan Province, PRC |
| *Pseudomonas* sp.1-BT-11 | MH028959 | Root tissue of *Panax Notoginseng* from Tonghai County of Yunnan Province, PRC |
| *Lysobacter capsici* ZST1-2 | KR092191.1 | Rhizoshere soils of purple potato from Fuyuan County of Yunnan Province, PRC |
| *Lysobacter antibioticus*13-1 | MH084836 | Rhizosphere Soil of rice from Kunming City of Yunnan Province, PRC |
| *Lysobacter antibioticus*13-6 | NZ_JMTZ00000000.1 | Rhizosphere soil of konjac from Fuyuan County of Yunnan Province, PRC |
| *Lysobacter antibioticus* HY | MH084845 | Seed tuber of Amorphophallus konjac from Fuyuan County of Yunnan Province, PRC |
