# Supplementary material for: Probiotic Consortia: Reshaping the Rhizospheric Microbiome and Its Role in Suppressing Root-Rot Disease of Panax notoginseng
Source: Front Microbiol. 2020 Apr 30;11:701. doi: 10.3389/fmicb.2020.00701 (PMC7203884; doi:10.3389/fmicb.2020.00701)
Supplement: TABLE S2 — Four probiotic consortia assembled by 13 different bacteria strains. [file Table_2.DOCX]

**Table S2. Four probiotic consortia assembled by 13 different bacteria strains**

| Groups | Strains | Composition of consortia assembled |
| --- | --- | --- |
| A | 13-1, 13-6, HY | 3-strains（*L. antibioticus*） |
| B | 1-JKT-10, 1-BT-11, ZST1-2, SQ-5 | 4-strains (*B. subtilis*, *P.* sp, *L. capsici*, *B. velezensis*) |
| C | C3, R2-2, SQ-5, B908 | 4-strains (*B. amyloliquefaciens*, *B. methylotrophicus*, *B. velezensis*, *B. subtilis*) |
| D | C3, R2-2, YQ-11, SQ-5, B908, M3, 1-JKT-10, 2-B-39 | 8-strains (2*B. amyloliquefaciens*, 2*B. velezensis*,  3*B. subtilis*, *B. methylotrophicus*) |
