# Supplementary material for: Probiotic Consortia: Reshaping the Rhizospheric Microbiome and Its Role in Suppressing Root-Rot Disease of Panax notoginseng
Source: Front Microbiol. 2020 Apr 30;11:701. doi: 10.3389/fmicb.2020.00701 (PMC7203884; doi:10.3389/fmicb.2020.00701)
Supplement: TABLE S3 — Suppression effects on diseased Panax notoginseng. [file Table_3.DOCX]

**Table S3. Suppression effects on diseased *Panax notoginseng***

| Treatment^a^ | Death rate (%) ± SE^b^ | Death rate reduction /% |
| --- | --- | --- |
| A | 42.47±2.71^*^ | 22.11 |
| B | 40.46±5.72^*^ | 24.12 |
| C | 54.60±4.37 | 9.98 |
| D | 31.49±4.32^*^ | 33.09 |
| E | 39.96±9.64^*^ | 24.62 |
| BT | 64.58±8.96 | - |

**Note:** a. A, B, C, and D represents 4 probiotic consortia, E represents biopesticide. BT represents severe diseased plants (treated with water), as control. b. Means and standard errors (SE) are shown. Values shown here with Tukey’s test at a *p*-value <0.05 marked as *.
