# Supplementary material for: Probiotic Consortia: Reshaping the Rhizospheric Microbiome and Its Role in Suppressing Root-Rot Disease of Panax notoginseng
Source: Front Microbiol. 2020 Apr 30;11:701. doi: 10.3389/fmicb.2020.00701 (PMC7203884; doi:10.3389/fmicb.2020.00701)
Supplement: TABLE S4 — Inhibition effects of the probiotic consortia against root-rot disease and quality evaluation of Panax notoginseng. [file Table_4.DOCX]

**Table S4. Inhibition effects of the** **probiotic consortia against root-rot disease and quality evaluation of *Panax notoginseng***

| Treatments ^a^ | Root length (cm) ± SE ^b^ | Root fresh weight (g) ± SE | Root dry weight (g) ± SE |
| --- | --- | --- | --- |
| A | 15.73±3.28 | 19.33±2.91^*^ | 5.78±0.33 |
| B | 12.67±2.99 | 17.47±2.53^*^ | 7.5±0.38 |
| C | 16.78±2.22 | 16.33±2.03 | 6.22±0.67 |
| D | 14.00±2.11 | 16.00±1.53 | 7.35±1.11 |
| E | 16.12±0.92 | 17.77±1.51^*^ | 6.25±1.41 |
| CK | 11.03±1.82 | 10.50±0.57 | 4.88±0.85 |

**Note:** a. A, B, C, and D represents 4 probiotic consortia, E represents biopesticide, and JKT represents control without any treatment. b. Means and standard errors (SE) are shown. Values shown here with Tukey’s test at a *p*-value < 0.05 marked as *.
