# Supplementary material for: Probiotic Consortia: Reshaping the Rhizospheric Microbiome and Its Role in Suppressing Root-Rot Disease of Panax notoginseng
Source: Front Microbiol. 2020 Apr 30;11:701. doi: 10.3389/fmicb.2020.00701 (PMC7203884; doi:10.3389/fmicb.2020.00701)
Supplement: TABLE S5 — Five major saponins (R1, Rg1, Re, Rb1, and Rd) in P. notoginseng. [file Table_5.DOCX]

**Table S5.** **Five major saponins (R1, Rg1, Re, Rb1, and Rd) in *P. notoginseng***

| Treatments ^a^ | R1(ppm)±SE | Rg1(ppm)±SE | Re(ppm)±SE | Rb1(ppm)±SE | Rd(ppm)±SE |
| --- | --- | --- | --- | --- | --- |
| A | 451978.16±101449.29** | 6490.66±274.74** | 776.93±14.81* | 8779.32±356.85** | 2951.95±314.45** |
| B | 414161.60±76804.39** | 6744.67±365.14** | 2128.62±122.23** | 9554.31±201.10** | 3256.67±286.43** |
| C | 507050.77±51339.189** | 6364.50±279.43** | 2042.75±394.65** | 7965.01±456.17** | 2615.92±104.22** |
| D | 486928.56±32768.37** | 7070.37±88.96** | 2019.45±300.59** | 6466.22±264.25** | 2128.54±58.53** |
| E | 196289.7±19362.60 | 6273.36±198.14** | 1678.68±157.61** | 9531.19±637.82** | 2810.66±95.62** |
| BT | 36038.22±2023.53 | 2424.58±251.80 | 133.80±2.53 | 1280.87±50.71 | 269.05±11.12 |
| JKT | 304553±22195.76* | 5479.60±220.29** | 1846.77±97.09** | 9529.36±322.38** | 2955.11±58.14** |

**Note: a.** A, B, C, and D represents 4 probiotic consortia, E represents biopesticide. JKT represents light diseased plants, BT represents severe diseased plants, both were treated with water; **b**. Means and standard errors (SE) are shown. Values shown here with LSD test at a *p*-value <0.05 marked as * and a *p*-value <0.01 marked as **.
