# Supplementary material for: Probiotic Consortia: Reshaping the Rhizospheric Microbiome and Its Role in Suppressing Root-Rot Disease of Panax notoginseng
Source: Front Microbiol. 2020 Apr 30;11:701. doi: 10.3389/fmicb.2020.00701 (PMC7203884; doi:10.3389/fmicb.2020.00701)
Supplement: TABLE S6 — Diversity indices of bacterial and fungal communities in rhizospheric soil. [file Table_6.DOCX]

**Table S6.** **Diversity indices of bacterial and fungal communities in rhizospheric soil**

| Types | Sample name | Abbreviation | Chao1  (97%) | OTU  (97%) | Shannon  (97%) | Simpson  (97%) |
| --- | --- | --- | --- | --- | --- | --- |
| Bacterial | rhizospheric soil treated by A probiotic consortia | A | 1323.76±83.16^a^ | 1051.33±40.62^ab^ | 7.92±0.09^ab^ | 0.99±0.15^a^ |
|  | rhizospheric soil treated by B probiotic consortia | B | 1135.95±29.59^ab^ | 853.33±56.05^b^ | 6.96±0.47^bc^ | 0.99±0.00^a^ |
|  | rhizospheric soil treated by C probiotic consortia | C | 1254.18±58.72^a^ | 984.33±22.67^ab^ | 7.74±0.12^ab^ | 0.97±0.01^a^ |
|  | rhizospheric soil treated by D probiotic consortia | D | 1311.79±11.06^a^ | 996.00±18.04^ab^ | 7.54±0.13^ab^ | 0.98±0.00^a^ |
|  | rhizospheric soil treated by E biopesticide | E | 1259.81±70.13^a^ | 1010.33±67.98^ab^ | 7.98±0.12^ab^ | 0.98±0.00^a^ |
|  | rhizospheric soil of light diseased plant | JKT | 1425.81±97.44^a^ | 1172.33±75.90^a^ | 8.41±0.25^a^ | 0.97±0.00^a^ |
|  | rhizospheric soil of severe diseased plant | BT | 914.44±30.99^b^ | 656.00±15.72^c^ | 6.54±0.19^c^ | 0.99±0.00^a^ |
| Fungal | rhizospheric soil treated by A probiotic consortia | A | 545.73±64.53^a^ | 465.33±48.77^a^ | 4.51±0.37^a^ | 0.78±0.047^a^ |
|  | rhizospheric soil treated by B probiotic consortia | B | 514.06±39.76^ab^ | 441.00±20.65^a^ | 4.344±0.14^a^ | 0.79±0.04^a^ |
|  | rhizospheric soil treated by C probiotic consortia | C | 591.37±27.13^a^ | 506.33±18.21^a^ | 5.10±0.26^a^ | 0.86±0.03^a^ |
|  | rhizospheric soil treated by D probiotic consortia | D | 557.19±20.53^a^ | 462.33±35.41^a^ | 4.33±0.34^a^ | 0.79±0.04^a^ |
|  | rhizospheric soil treated by E biopesticide | E | 576.52±73.26^a^ | 505.00±66.02^a^ | 5.21±0.71^a^ | 0.87±0.06^a^ |
|  | rhizospheric soil of light diseased plant | JKT | 487.27±14.82^ab^ | 444.67±25.01^a^ | 5.42±0.30^a^ | 0.92±0.01^a^ |
|  | rhizospheric soil of severe diseased plant | BT | 333.22±43.12^b^ | 229.00±35.93^b^ | 2.34±0.28^b^ | 0.56±0.09^b^ |

1. **Means and standard errors (SE) are shown. b. Values shown here** **with Tukey’s test at p<0.05.**
