# Supplementary material for: Probiotic Consortia: Reshaping the Rhizospheric Microbiome and Its Role in Suppressing Root-Rot Disease of Panax notoginseng
Source: Front Microbiol. 2020 Apr 30;11:701. doi: 10.3389/fmicb.2020.00701 (PMC7203884; doi:10.3389/fmicb.2020.00701)
Supplement: TABLE S7 — Relative abundance at the phylum level in bacterial communities. [file Table_7.DOCX]

**Table S7. Relative abundance at the phylum level in bacterial communities**

| Bacterial | **A** | **B** | **C** | **D** | **E** | **JKT** | **BT** |
| --- | --- | --- | --- | --- | --- | --- | --- |
| Proteobacteria | 0.574±0.006 | 0.691±0.100 | 0.537±0.084* | 0.606±0.026 | 0.601±0.065 | 0.480±0.051** | 0.683±0.086 |
| Acidobacteria | 0.215±0.007** | 0.139±0.066* | 0.193±0.098** | 0.163±0.029* | 0.219±0.054** | 0.229±0.063** | 0.039±0.004 |
| Bacteroidetes | 0.081±0.005* | 0.083±0.024* | 0.101±0.049* | 0.107±0.050 | 0.067±0.015** | 0.106±0.029 | 0.175±0.074 |
| Actinobacteria | 0.049±0.010** | 0.037±0.018 | 0.048±0.005** | 0.046±0.012* | 0.050±0.007** | 0.069±0.031** | 0.011±0.003 |
| Gemmatimonadetes | 0.040±0.003** | 0.021±0.005* | 0.027±0.016** | 0.031±0.004** | 0.025±0.004* | 0.056±0.016** | 0.004±0.001 |
| Firmicutes | 0.007±0.005 | 0.006±0.002 | 0.067±0.102 | 0.019±0.015 | 0.004±0.002 | 0.010±0.001 | 0.081±0.104 |
| Nitrospirae | 0.008±0.001 | 0.005±0.003 | 0.009±0.009 | 0.008±0.001 | 0.007±0.001 | 0.011±0.004* | 0.002±0.001 |
| Verrucomicrobia | 0.005±0.002* | 0.002±0.001 | 0.003±0.002 | 0.004±0.001 | 0.007±0.003** | 0.008±0.003** | 0.001±0.001 |
| Elusimicrobia | 0.004±0.001** | 0.002±0.001 | 0.002±0.002 | 0.002±0.000 | 0.002±0.002 | 0.006±0.003** | 0.000±0.000 |
| Saccharibacteria | 0.002±0.000* | 0.003±0.001** | 0.001±0.001 | 0.002±0.000* | 0.004±0.002** | 0.001±0.000 | 0.000±0.000 |
| TM6 | 0.002±0.001 | 0.002±0.002 | 0.002±0.001 | 0.002±0.002 | 0.002±0.000 | 0.002±0.001 | 0.001±0.000 |
| Chlorobi | 0.003±0.000** | 0.001±0.001 | 0.001±0.001 | 0.002±0.000** | 0.002±0.001** | 0.004±0.000** | 0.000±0.000 |
| Tenericutes | 0.001±0.001 | 0.001±0.000 | 0.002±0.002 | 0.001±0.000 | 0.000±0.000 | 0.005±0.008 | 0.000±0.000 |
| Chloroflexi | 0.001±0.000* | 0.001±0.001 | 0.001±0.000* | 0.001±0.001* | 0.002±0.001** | 0.001±0.000* | 0.000±0.000 |
| Parcubacteria | 0.001±0.000* | 0.001±0.000* | 0.001±0.000* | 0.001±0.000** | 0.001±0.000** | 0.002±0.000** | 0.000±0.000 |

**Note:** Relative abundance at the phylum level in bacterial communities of the top fifteen species. Values shown here with Tukey’s test at a p-value <0.05 marked as * and a p-value <0.01 marked as **.
