# Supplementary material for: Probiotic Consortia: Reshaping the Rhizospheric Microbiome and Its Role in Suppressing Root-Rot Disease of Panax notoginseng
Source: Front Microbiol. 2020 Apr 30;11:701. doi: 10.3389/fmicb.2020.00701 (PMC7203884; doi:10.3389/fmicb.2020.00701)
Supplement: TABLE S8 — Classification according to the taxonomic units of phylum (abundance > 1%). [file Table_8.DOCX]

**Table S8. Classification according to the taxonomic units of phylum (abundance > 1%)**

| Type | Phylum | P^b^ | ^a^A/% | B/% | C/% | D/% | E/% | JKT/% | BT/% |
| --- | --- | --- | --- | --- | --- | --- | --- | --- | --- |
| Bacterial | Acidobacteria | 0.05 | 21.52 | 13.93 | 19.29 | 16.31 | 21.92 | 22.90 | 3.89 |
|  | Actinobacteria | 0.14 | 4.87 | 3.65 | 4.81 | 4.59 | 5.02 | 6.91 | 1.09 |
|  | Bacteroidetes | 0.18 | 8.09 | 8.32 | 10.09 | 10.71 | 6.67 | 10.58 | 17.54 |
|  | Firmicutes | 0.04 | - | - | 6.67 | 1.92 | - | - | 8.05 |
|  | Gemmatimonadetes | 0.02 | 3.97 | 2.12 | 2.69 | 3.05 | 2.48 | 5.56 | - |
|  | Nitrospirae | 0.10 | - | - | - | - | - | 1.09 | - |
|  | Proteobacteria | 0.05 | 57.40 | 69.11 | 53.74 | 60.56 | 60.09 | 48.03 | 68.34 |
| Fungal | Ascomycota | 0.03 | 28.14 | 32.31 | 38.63 | 30.40 | 36.19 | 61.54 | 96.40 |
|  | Basidiomycota | 0.07 | 4.31 | 3.75 | 4.65 | 7.00 | 24.93 | 5.89 | - |
|  | Glomeromycota | 0.03 | 4.81 | 2.01 | 3.18 | 1.42 | 3.30 | - | - |
|  | Rozellomycota | 0.05 | - | 1.07 | 1.13 | 2.64 | 1.80 | 1.18 | - |
|  | Unidentified | 0.11 | 7.93 | 9.93 | 16.22 | 6.54 | 10.98 | 6.32 | 1.12 |
|  | Zygomycota | 0.02 | 53.35 | 49.91 | 35.15 | 51.04 | 22.12 | 24.13 | 1.88 |

1. A, B, C, and D represents 4 probiotic consortia, E represents biopesticide, JKT represents light diseased plants (treated with water), BT represents severe diseased plants (treated with water）as control. **b**. Values shown here with Tukey’s test at a *p*-value <0.05
