# Supplementary material for: Probiotic Consortia: Reshaping the Rhizospheric Microbiome and Its Role in Suppressing Root-Rot Disease of Panax notoginseng
Source: Front Microbiol. 2020 Apr 30;11:701. doi: 10.3389/fmicb.2020.00701 (PMC7203884; doi:10.3389/fmicb.2020.00701)
Supplement: TABLE S9 — Comparison and analysis of the bacteria genera in rhizospheric soil of light diseased plants and severe diseased plants. [file Table_9.DOCX]

**Table S9. Comparison and analysis of the bacteria genera in rhizospheric soil of light diseased plants and severe diseased plants**

| Type | Bacterial genera | JKT/ % | BT/ % | Difference value |
| --- | --- | --- | --- | --- |
| Dominant bacterial genera in rhizospheric soil of healthy plants | *Ambiguous taxa* | 1.61 | 0.32 | 1.28 |
|  | *Acidibacter* | 2.34 | 0.25 | 2.09 |
|  | *Bryobacter* | 2.42 | 0.38 | 2.03 |
|  | *Candidatus Solibacter* | 3.20 | 0.39 | 2.81 |
|  | *Gemmatimonas* | 3.00 | 0.15 | 2.85 |
|  | *Haliangium* | 1.43 | 0.09 | 1.34 |
|  | *Other* | 24.50 | 20.26 | 4.24 |
|  | *Rhizomicrobium* | 2.05 | 0.82 | 1.23 |
|  | *Rhodanobacter* | 1.32 | 0.23 | 1.09 |
|  | *Uncultured* | 21.92 | 5.75 | 16.18 |
|  | *Uncultured bacterium* | 3.31 | 0.65 | 2.66 |
|  | *Uncultured gamma proteobacterium* | 1.74 | 0.21 | 1.53 |
| Dominant bacterial genera in rhizospheric soil of diseased plants | *Aeromonas* | 0.00 | 1.70 | 1.70 |
|  | *Chryseobacterium* | 0.01 | 2.25 | 2.25 |
|  | *Clostridium sensu stricto 1* | 0.06 | 7.23 | 7.17 |
|  | *Enterobacter* | 0.00 | 5.61 | 5.61 |
|  | *Flavobacterium* | 0.05 | 5.96 | 5.92 |
|  | *Mucilaginibacter* | 0.89 | 2.17 | 1.28 |
|  | *Novosphingobium* | 0.05 | 1.60 | 1.55 |
|  | *Pseudomonas* | 0.09 | 2.30 | 2.21 |
|  | *Rhizobium* | 0.13 | 1.34 | 1.21 |
|  | *Ramlibacter* | 0.07 | 1.34 | 1.27 |
|  | *Sphingomonas* | 3.71 | 5.27 | 1.55 |
|  | *Sphingobacterium* | 0.00 | 1.92 | 1.92 |
|  | *Stenotrophomonas* | 0.01 | 4.37 | 4.37 |
|  | *Sphingobium* | 0.12 | 6.78 | 6.66 |

**Note:** The genera of bacteria listed in the table as the difference value of relative abundance (> 1%) of light diseased plants and severe diseased plants.
