# Supplementary material for: Probiotic Consortia: Reshaping the Rhizospheric Microbiome and Its Role in Suppressing Root-Rot Disease of Panax notoginseng
Source: Front Microbiol. 2020 Apr 30;11:701. doi: 10.3389/fmicb.2020.00701 (PMC7203884; doi:10.3389/fmicb.2020.00701)
Supplement: TABLE S10 — Relative abundance at the phylum level in fungal communities. [file Table_10.DOCX]

**Table S10. Relative abundance at the phylum level in fungal communities**

| Fungal | **A** | **B** | **C** | **D** | **E** | **JKT** | **BT** |
| --- | --- | --- | --- | --- | --- | --- | --- |
| Ascomycota | 0.281±0.061** | 0.323±0.058** | 0.386±0.065** | 0.304±0.094** | 0.362±0.202** | 0.615±0.054** | 0.964±0.022 |
| Zygomycota | 0.534±0.094** | 0.499±0.116** | 0.352±0.163** | 0.510±0.149** | 0.221±0.115** | 0.241±0.029** | 0.019±0.017 |
| Unidentified | 0.079±0.033 | 0.099±0.083 | 0.162±0.169** | 0.065±0.021 | 0.110±0.005 | 0.063±0.022 | 0.011±0.003 |
| Basidiomycota | 0.043±0.024 | 0.038±0.009 | 0.046±0.010 | 0.070±0.064 | 0.249±0.333** | 0.059±0.004 | 0.005±0.003 |
| Glomeromycota | 0.048±0.023** | 0.020±0.023 | 0.032±0.002 | 0.014±0.008 | 0.033±0.038 | 0.006±0.006 | 0.000±0.000 |
| Rozellomycota | 0.006±0.000 | 0.011±0.013 | 0.011±0.005 | 0.026±0.035 | 0.018±0.02 | 0.002±0.001 | 0.000±0.000 |
| Chytridiomycota | 0.007±0.004 | 0.009±0.009 | 0.009±0.008 | 0.009±0.009 | 0.006±0.003 | 0.012±0.005** | 0.000±0.000 |
| Cercozoa | 0.001±0.000 | 0.001±0.000 | 0.002±0.001 | 0.001±0.001 | 0.001±0.001 | 0.001±0.001 | 0.000±0.001 |
| Neocallimastigomycota | 0.000±0.000 | 0.000±0.000 | 0.000±0.000 | 0.000±0.000 | 0.000±0.000 | 0.000±0.000 | 0.000±0.000 |
| Blastocladiomycota | 0.000±0.000 | 0.000±0.000 | 0.000±0.000 | 0.000±0.000 | 0.000±0.000 | 0.000±0.000 | 0.000±0.000 |

**Note:** Relative abundance at the phylum level in fungal communities of the top ten species. Values shown here with Tukey’s test at a p-value <0.05 marked as * and a p-value <0.01 marked as **.
