# Supplementary material for: Probiotic Consortia: Reshaping the Rhizospheric Microbiome and Its Role in Suppressing Root-Rot Disease of Panax notoginseng
Source: Front Microbiol. 2020 Apr 30;11:701. doi: 10.3389/fmicb.2020.00701 (PMC7203884; doi:10.3389/fmicb.2020.00701)
Supplement: TABLE S11 — Comparison and analysis of the fungal genera in rhizospheric soil of lightly diseased plants and severely diseased plants. [file Table_11.DOCX]

**Table S11. Comparison and analysis of the fungal genera in rhizospheric soil of light diseased plants and severe diseased plants**

| Type | Fungal genera | JKT/% | BT/% | Difference value |
| --- | --- | --- | --- | --- |
| Dominant fungal genera in rhizospheric soil of healthy plants | *Cladophialophora* | 1.19 | 0.14 | 1.05 |
|  | *Leptodontidium* | 3.67 | 0.10 | 3.57 |
|  | *Minimedusa* | 1.18 | 0.03 | 1.15 |
|  | *Mortierella* | 2.46 | 0.30 | 2.16 |
|  | *Solicoccozyma* | 1.05 | 0.04 | 1.01 |
|  | *Trichoderma* | 5.49 | 0.09 | 5.40 |
|  | *Unidentified genus* | 58.29 | 15.22 | 43.07 |
| Dominant fungal genera in rhizospheric soil of diseased plants | *Clonostachys* | 0.12 | 62.73 | 62.60 |
|  | *Exophiala* | 0.47 | 12.42 | 11.94 |
|  | *Fusarium* | 2.35 | 13.11 | 10.76 |
|  | *Xylaria* | 0.80 | 11.47 | 10.67 |

**Note:** The genera of fungi listed in the table as the difference value of relative abundance (> 1%) of light diseased plants and severe diseased plants.
